# Racial Differences in Expression Levels of miRNA Machinery-Related Genes, Dicer, Drosha, DGCR8, and AGO2, in Asian Korean Papillary Thyroid Carcinoma and Comparative Validation Using the Cancer Genome Atlas

**DOI:** 10.1155/2017/5789769

**Published:** 2017-03-02

**Authors:** Jaegil Kim, Woo-Jae Park, Kwang-Joon Jeong, Sun Hee Kang, Sun Young Kwon, Shin Kim, Jong-Wook Park

**Affiliations:** ^1^The Eli and Edythe L. Broad Institute of Massachusetts Institute of Technology and Harvard University, Cambridge, MA, USA; ^2^Department of Biochemistry, School of Medicine, Gachon University, Incheon 21936, Republic of Korea; ^3^Department of Microbiology, Chonnam National University Medical School, 160, Baekseo-ro, Dong-gu, Gwangju 61469, Republic of Korea; ^4^Department of Surgery, School of Medicine, Keimyung University, 1095 Dalgubeol-daero, Dalseo-gu, Daegu 42601, Republic of Korea; ^5^Department of Pathology, School of Medicine, Keimyung University, 1095 Dalgubeol-daero, Dalseo-gu, Daegu 42601, Republic of Korea; ^6^Department of Immunology, School of Medicine & Institute of Medical Science, Keimyung University, 1095 Dalgubeol-daero, Dalseo-gu, Daegu 42601, Republic of Korea

## Abstract

Aberrant regulation of microRNA (miRNA) machinery components is associated with various human cancers, including papillary thyroid carcinoma (PTC), which is the most common type of thyroid cancer, and a higher prevalent female malignancy. The purpose of this study is to investigate racial differences in mRNA expression levels of four miRNA machinery components, Dicer, Drosha, DGCR8, and AGO2, and their correlations with clinicopathological characteristics. Forty PTC samples from female Asian Korean PTC patients were enrolled. Using qPCR, we examined mRNA expression levels of the components and next validated our results by comparison with results of female white American in the TCGA PTC project. Interestingly, mRNA expression levels of the selected factors were altered in the TCGA PTC samples. However, only Drosha showed a significantly lower expression level in Asian Korean PTC samples. Furthermore, the mRNA expression levels of the four components showed no association with clinicopathological characteristics in both groups. On the other hand, positive correlations were observed between altered mRNA expression levels of Dicer and Drosha and DGCR8 and Drosha in TCGA PTC samples. These findings collectively revealed that altered mRNA expression levels of miRNA machinery components might be responsible for racial differences in the carcinogenesis of PTC.

## 1. Introduction 

Thyroid cancer is the most common endocrine malignancy in the head and neck region. One of the major histopathologic types is papillary thyroid carcinoma (PTC), which accounts for approximately 85% of all thyroid cancers [1]. In contrast to women in the United States, the United Kingdom, and Japan, PTC is the most prevalent type of thyroid carcinoma among Korean women [2]. This is a very important issue because racial differences in the genetic factors of PTC could influence carcinogenesis or clinicopathological characteristics.

Only a small fraction of the genome consists of protein-coding genes and some RNAs are transcribed from many noncoding intergenic regions. MicroRNA (miRNA) is one of the several classes of noncoding RNA transcribed from intergenic region [3]. These transcribed miRNA molecules are processed by a well-organized processing mechanism, referred to as the “miRNA machinery” [4], which is controlled by various components such as RNase III endonuclease Drosha (RNASEN), a double-strand RNA binding protein called the DiGeorge syndrome critical region gene 8 (DGCR8), the RNase III ribonuclease Dicer, and a component of the RNA-induced silencing complex (RISC) called Argonaute 2 (AGO2). During the biogenesis of miRNA, Drosha cleaves the primary miRNAs in the nucleus, resulting in precursor-miRNAs (pre-miRNAs) that are further processed in the cytoplasm by Dicer to generate mature miRNA molecules. Mature miRNAs regulate gene expression at the posttranscriptional level by translational repression and/or cleavage of the target mRNA [5].

The regulation of miRNA biogenesis must be finely tuned and well balanced because dysregulation of the miRNA machinery affects pathophysiologies of various diseases [6–11]. Among these diseases, aberrant expression levels of miRNA machinery components are implicated in carcinogenesis. For example, Dicer, the key enzyme in the miRNA machinery, is dysregulated in acute myeloid leukemia [12], hepatocellular carcinoma [13], invasive ductal breast carcinoma [14], ovarian serous carcinoma [15], pleomorphic adenomas of the salivary gland [16], and prostate adenocarcinoma [17]. Additionally, Drosha, a part of the microprocessor complex, is dysregulated in cervical squamous cell carcinoma [18], colorectal carcinoma [19], endometrial cancers [20], and metastatic serous ovarian carcinoma [21]. Moreover, DGCR8, which stabilizes Drosha through direct interaction, is dysregulated in prostate cancer [22], colorectal carcinoma [23], and epithelial skin cancer [24]. Lastly, AGO2, a component of the RNA-induced silencing complex, is dysregulated in epithelial skin cancer [24] and invasive breast carcinoma [25]. These results revealed that an aberration in the expression of components of the miRNA processing machinery (Dicer, Drosha, DGCR8, and AGO2) might be correlated with pathophysiology of cancers. Our previous study showed that mRNA expression levels of Drosha were significantly decreased in Asian Korean PTC tissues and that mRNA expression levels of Dicer and AGO2 were positively correlated with each other, suggesting that Drosha might play an important role in development of PTC of Asian Koreans and the two components, Dicer and AGO2, may share partially common regulating mechanisms in PTC of Asian Koreans [26].

The Cancer Genome Atlas (TCGA) project includes the largest cohort studies for comprehensive multiplatform analysis of various cancers. The TCGA data portal contains information about DNA (mutation, copy number, and methylation), RNA (transcript levels), and proteins (abundance and phosphorylation) in various cancers [27]. However, TCGA project data almost come from various racial patients, except Asian Koreans. Moreover, mRNA expression levels of miRNA machinery components and their clinicopathological correlations have not been evaluated in the TCGA data sets of papillary thyroid carcinoma [28].

In the present study, we investigated the expression levels of miRNA machinery components and analyzed their correlations with clinicopathological characteristics, including age, histologic stage, T stage, N stage, M stage, extrathyroidal extension, bilateral lobe involvement, and multifocality in Asian Korean PTC samples and white American data from the TCGA database. Furthermore, we evaluated racial differences between the results from analysis of Asian Korean samples and white American PTC patients in the TGCA database.

## 2. Materials and Methods

### 2.1. Patients and Tissues

A total of 40 female patients who were diagnosed with PTC were included in the study. All patients were of Asian race and the mean age was 46.33 ± 14.35 years. This series of tumor and nonneoplastic samples was used in our previous studies [26]. Papillary thyroid carcinomas and adjacent nonneoplastic tissues were obtained from patients undergoing surgery in Dongsan Medical Center (Daegu, Korea) between July 2008 and December 2011. Tissue samples were immediately frozen in liquid nitrogen and stored at −80°C until RNA isolation. Tissue samples were provided from the Keimyung Human Bio-Resource Bank, Korea. The purpose of the study was explained to each patient and informed consent was obtained from each study participant. The protocols were approved by the Institutional Review Board of Keimyung University Dongsan Medical Center (approval #12-41).

### 2.2. Gene Expression Databases

The four miRNA machinery component genes present in the TCGA RNA-Seq profiles were DICER1, RNASEN (Drosha), DGCR8, and EIF2C2 (AGO2). RNA-Seq analysis was followed by the RSEM normalization method. Raw gene expression data for a sixth cohort were downloaded from The Cancer Genome Atlas (TCGA; https://cancergenome.nih.gov). The full data for this cohort were described in detail previously [28]. Comprehensive genetic information was available for 496 patients with primary PTC in the cohort. Among the total data, we selected 31 paired data sets with expression level data for the four miRNA machinery components in PTC and adjacent nonneoplastic tissues from white PTC patients because only 31 cases had available data sets for comparative study in the present study. This study meets the publication guidelines provided by TCGA.

### 2.3. RNA and Quantitative Real-Time RT-PCR

Total cellular RNA was extracted from tissues using TRIzol reagent (Molecular Research Center, Inc., Cincinnati, OH, USA). RNA was quantified using NanoDrop 1000 (Thermo Scientific, Wilmington, USA). Each cDNA was synthesized from 2 *μ*g of total RNA using MMLV reverse transcriptase (Promega, Madison, WI, USA) according to the manufacturer's protocol. Quantitative real-time PCR (qPCR) was performed on the LightCycler® 480 real-time PCR system (Roche Diagnostics, Mannheim, Germany) using the specific primer pairs described in Table 1 and SYBR Green Premix (Toyobo, Japan). *β*-Actin was used as a housekeeping gene for normalization, and a no-template sample was used as a negative control.

### 2.4. Statistics

Statistical analysis was performed with SPSS 19.0 (SPSS Inc., Chicago, IL, USA). Statistical comparisons for significance were made with Wilcoxon signed-rank test for paired samples. The mean value was used as cut-off value (low versus high). Clinicopathological implications of the mRNA expression levels of miRNA biogenesis-related components were analyzed using linear-by-linear association, Pearson's Chi-squared test, and Fisher's exact test for categorical variables. Differences in TCGA data between the groups were analyzed using Student's *t*-test or Mann–Whitney *U* test. Correlations between relative mRNA expressions of individual miRNA machinery components in TCGA PTC data were analyzed by Pearson's correlation coefficient analysis. A *P* value less than 0.05 was considered to denote significance for all statistical analyses performed in this study.

## 3. Results

### 3.1. Expression Levels of miRNA Machinery Components in PTC

**Figure 1:**
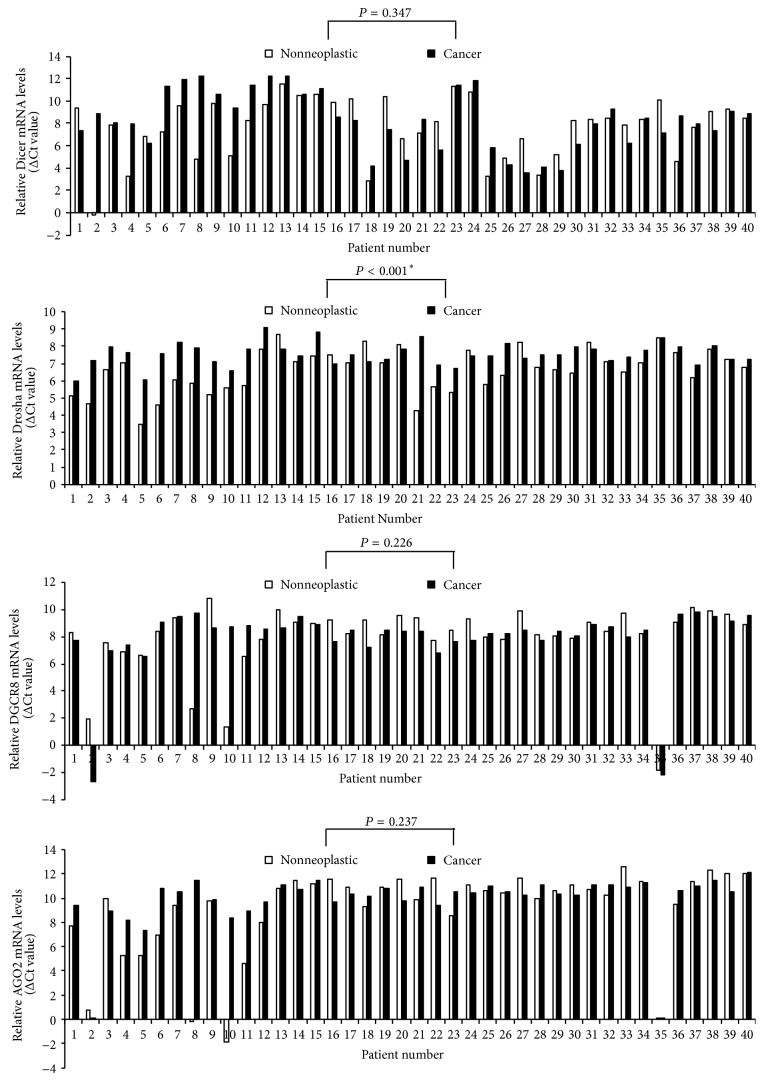
Relative mRNA levels of Dicer, Drosha, DGCR8, and AGO2 (normalized to the corresponding *β*-actin mRNAs) in Asian Korean PTC tissues compared with adjacent noncancerous thyroid tissues.* Asterisk* indicates Wilcoxon signed-rank test.

**Figure 2:**
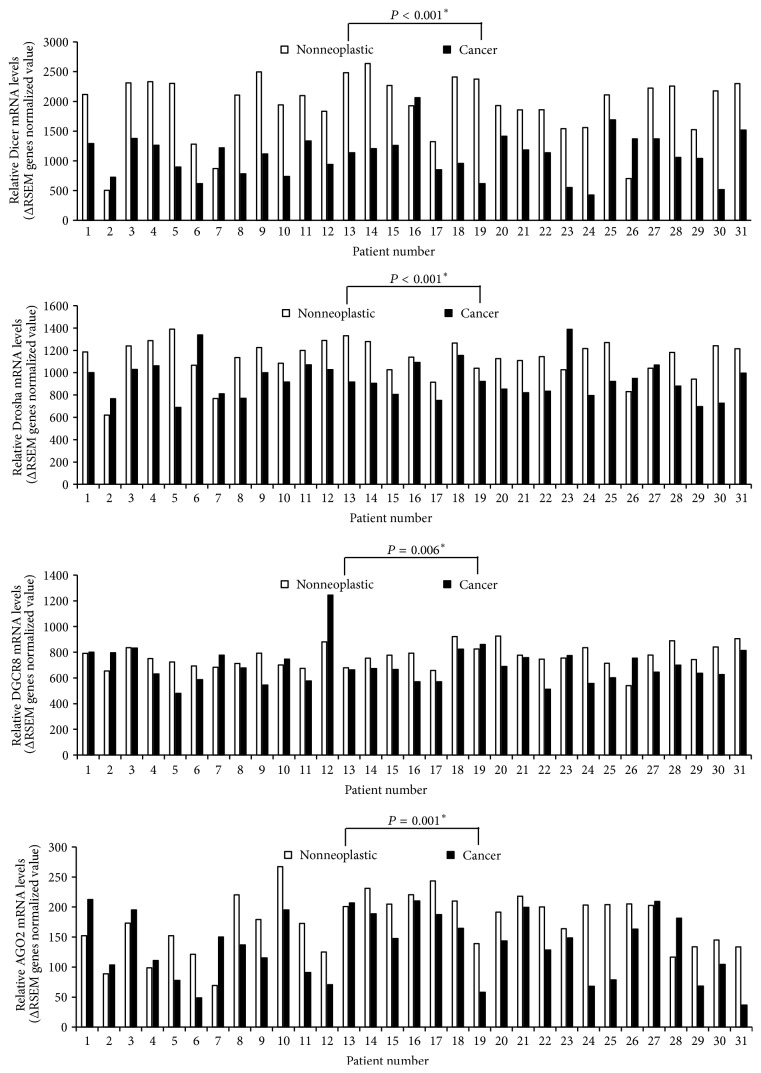
Relative mRNA levels of Dicer, Drosha, DGCR8, and AGO2 in TCGA PTC data.* Asterisk* indicates Wilcoxon signed-rank test.

**Figure 3:**
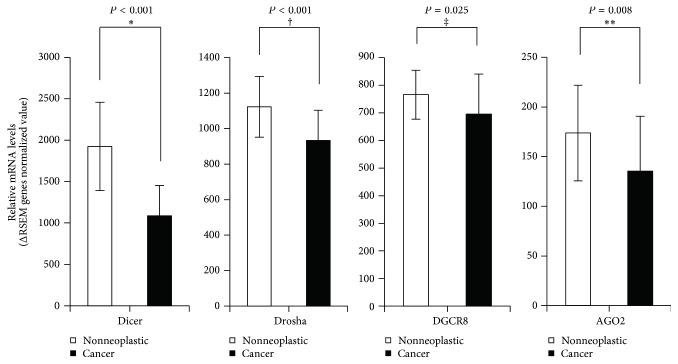
Relative mRNA expressions of Dicer, Drosha, DGCR8, and AGO2 in TCGA PTC data; ^*∗*^*P* < 0.001, ^†^*P* < 0.001, ^‡^*P* = 0.025, and ^*∗∗*^*P* = 0.008.* Asterisk* indicates Mann–Whitney *U* test. Dagger and double dagger indicate Student's *t*-test.

Expression levels of four miRNA machinery components were examined for racial differences. Firstly, we took samples from cancer and adjacent noncancer tissues from 40 PTC patients of Asian Korean origin. Then, we quantified mRNA expression levels of miRNA machinery components by qPCR of paired samples of 40 Asian Korean patients with PTC; *β*-actin mRNA levels were used for normalization of each sample. As shown in Figure 1, mRNA expression levels of Drosha showed a significant difference; malignant tissues showed a comparatively lower mRNA expression level as compared to the nonneoplastic tissues in a total of 31 among 40 Asian Korean patients with PTC corresponding to 77.5% (*P* < 0.001). Secondly, we took cancer genomics data sets of 31 white American PTC patients from TCGA through the cBioPortal for Cancer Genomics (cBioPortal) (http://www.cbioportal.org/). As shown in Figure 2, the analyzed TCGA PTC data using various analytic methods showed that Dicer, Drosha, DGCR8, and AGO2 mRNA expression levels were found to be significantly lower in carcinomatous tissues as compared to the nonneoplastic tissues in white American patients with PTC (Dicer: *P* < 0.001; Drosha: *P* < 0.001; DGCR8: *P* = 0.006; AGO2: *P* = 0.001). Further, the mean mRNA expression levels in PTC tissues were significantly lower compared to those of nonneoplastic thyroidal tissues (Dicer: *P* < 0.001; Drosha: *P* < 0.001; DGCR8: *P* = 0.025; AGO2: *P* = 0.008; Figure 3).

### 3.2. Relationship between mRNA Expression Levels of Individual miRNA Machinery Components in PTC

**Figure 4:**
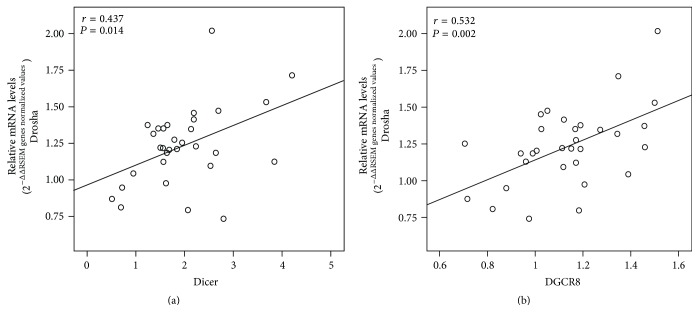
Correlation between mRNA expression levels of individual components in TCGA PTC data. Correlations were found between Dicer and Drosha and between Drosha and DGCR8; ^*∗*^*P* = 0.014 and ^*∗∗*^*P* = 0.002, respectively.

Earlier, we have shown that changes in the mRNA expression levels of AGO2 and Dicer exhibited a positive correlation in female Asian Korean patients with PTC [26]. Hence, we checked for correlations in mRNA levels of four miRNA machinery components in the 31 white American PTC patients from TCGA PTC data. Our results showed association between Drosha and Dicer and also between DGCR8 and Drosha with Pearson's correlation coefficient value of 0.437 (*P* = 0.014) and 0.532 (*P* = 0.002), respectively (Figure 4).

### 3.3. Relationship between mRNA Expression Levels of Individual miRNA Machinery Components and Clinical Parameters of Patients with PTC

The influence of clinical parameters on mRNA expression levels of miRNA machinery components was evaluated by classifying patients according to each clinical characteristic. Before statistical analysis, the raw qPCR data of mRNA expression levels were calculated by using the 2^−ΔΔCt^ method [29].  Table 2 shows clinicopathological characteristics of 40 Asian Korean PTC patients (mean age: 46.33 ± 14.35 years). mRNA expression levels of the miRNA machinery components did not show significant statistical associations with age, histologic stage, T stage, N stage, M stage, extrathyroidal extension, bilateral lobe involvement, and multifocality in Asian Korean PTC specimens (Table 2). The clinicopathological characteristics of all 31 patients (mean age: 42.10 ± 15.29 years) from the TCGA PTC data set are presented in Table 3. Similarly, the mRNA expression levels were not associated with age, histologic stage, T stage, N stage, M stage, and multifocality in white American patients of the TCGA (Table 3).

## 4. Discussion

miRNAs are important regulators of gene expression via transcriptional cleavage and/or translational repression of their target mRNAs. Upregulation of specific oncomiRs or genetic deletion of tumor suppressor miRNAs is associated with human carcinogenesis [30, 31], suggesting that aberrant miRNA biogenesis plays an important role in the pathophysiology of cancer, including papillary thyroid carcinoma [26]. However, racial differences in the pathophysiology of PTC related to dysregulation of miRNA biogenesis components are poorly understood. Therefore, in this study, we aimed to determine the mRNA expression levels of miRNA machinery factors by RT-qPCR and their clinical significance in female Asian Korean patients with PTC and to validate the results in comparison with PTC TCGA data from white American patients. Notably, we found that expression levels of all of the selected factors were altered in TCGA PTC samples of female white Americans, but only Drosha expression was significantly lower in PTC tissues compared with nontransformed tissue in the Asian Korean patients.

Previous studies have reported significantly upregulated (specimens at Sapienza University of Rome, Italy, and the Institut Gustave Roussy, Villejuif, France [32]) or downregulated (specimens at New York Presbyterian Hospital-Weill Cornell Medical College [33]) levels of Dicer in thyroid carcinoma, significantly higher (specimens from St. James's Hospital, Dublin [15]) or lower (specimens at the University of Turin, Italy [34], and from the M.D. Anderson Cancer Center Tumor Bank and the Brigham and Women's Gynecologic Oncology Tumor Bank [11]) levels of Dicer and Drosha in ovarian carcinomas, and significantly higher (specimens at the Campus Bio-Medico University of Rome [19]) or no significant difference (specimens at the University Hospital of Patras, Greece [4]) in Drosha level in colorectal carcinoma. However, although the race of the patients enrolled in these studies was not precisely identified, the data suggested racial differences in the carcinogenesis of PTC. In the present study, we compared the mRNA expression levels of the miRNA machinery components in PTC tissue and adjacent nonneoplastic thyroid tissue in 40 Asian Korean PTC patients. Furthermore, we evaluated the expression levels of Dicer, Drosha, DGCR8, and AGO2 in 31 white American PTC patients with mRNA expression data from the TCGA database. Compared with adjacent nonneoplastic tissue, a wide variation in mRNA levels of miRNA machinery components was detected in the PTC samples. We found that only mRNA expression of Drosha was significantly lower in malignant tissues than in the corresponding nonneoplastic tissues in both racial groups (Figures 1 and 2). In contrast, the changes in mRNA expression profile of the other components differed between the racial groups. In the TCGA data, downregulated mRNA expression levels of each component were observed (Dicer, 27 of 31; DGCR, 23 of 31; AGO2, 23 of 31; Figure 2). These results indicated that mRNA expression levels of all four miRNA machinery components were downregulated in a large proportion of the PTC samples and suggested that downregulation of these four components might play an important role in carcinogenesis of PTC in white American patients. However, in Asian Korean PTC patients, only downregulation of Drosha appears to be involved in the carcinogenesis of PTC.

We previously reported that mRNA expression levels of Dicer and AGO2 are positively correlated in Asian Korean PTC samples [26]. In this study, we found significant positive correlations between Dicer and Drosha and between DGCR8 and Drosha in the TCGA PTC data. The nuclear RNase III endonuclease Drosha, the cytoplasmic RNase III ribonuclease Dicer, and the microprocessor component DGCR8 are essential for miRNA maturation [35]. In particular, Drosha and Dicer are crucial nucleases in the miRNA machinery [36, 37]. Moreover, Drosha and DGCR8 are components of a complicated double-negative feedback circuit in which DGCR8 directly interacts with Drosha, resulting in stabilization of Drosha [38]. Therefore, our data suggest that the correlations between Dicer and Drosha and between DGCR8 and Drosha are unique features in PTC progression of white American patients, in comparison with Asian Korean patients.

Recently, there has been accumulating evidence that dysregulation of miRNA biogenesis components is significantly implicated in the pathophysiology of a variety of cancers [21, 39–42]. However, there are few studies on the regulation of miRNA components and their correlation with clinicopathological features in PTC [33]. In the present study, we investigated the correlation between mRNA expression levels of important miRNA machinery components and clinicopathological characteristics in PTC, including age, histologic stage, T stage, N stage, M stage, extrathyroidal extension, bilateral lobe involvement, and multifocality. However, mRNA expression levels of miRNA machinery components were not statistically associated with any clinicopathological parameters in both Asian Korean samples and white American samples of the TCGA PTC data set (Tables 2 and 3).

To the best of our knowledge, this is the first study evaluating racial differences between Asian Korean and white American patients in the pathophysiology of PTC related to dysregulation of miRNA biogenesis. The causes of the observed racial disparities in the expression levels of miRNA machinery components in PTC are thought to be multifactorial and are probably affected by a complex mixture of genetic and/or epigenetic changes. Therefore, further studies on the clinicopathological significance of alterations in miRNA machinery components and their underlying mechanisms in the carcinogenesis of PTC are required.

Results of this study should be considered with caution because qPCR and RNA-Seq are different methods with different sensitivity, principle of experiment, and dynamic ranges. Nevertheless, in the present study, we provide interesting evidences for racial differences in the expression levels of miRNA machinery components and in the correlations between mRNA expression levels of individual miRNA machinery components between Asian Korean and white American patients with PTC. Taken together, these findings revealed for the first time that altered expression levels of miRNA machinery components might be responsible for racial differences in the carcinogenesis of PTC.

## 5. Conclusions

The regulation of miRNA biogenesis must be finely tuned and well balanced because dysregulation of the miRNA machinery affects pathophysiologies of various diseases, including cancer. Papillary thyroid carcinoma (PTC) is one of the major histopathologic types of thyroid cancer, which is the most common endocrine malignancy in the head and neck region. Genetic factors associated racial differences in prevalence of PTC have not been evaluated in the regulation of miRNA biogenesis components. The results of this study show that the expression levels of the selected factors were altered in the TCGA PTC samples of female white American patients. In contrast, only Drosha showed a significantly lower expression level in Asian Korean PTC tissues. Although, for both Asian Korean samples and white American PTC data of the TCGA, altered mRNA expression levels of the components showed no association with clinicopathological characteristics, positive correlations were observed between altered mRNA expression levels of Dicer and Drosha and between DGCR8 and Drosha in the TCGA data set. Taken together, the altered expression levels of miRNA machinery components might affect racial differences in the carcinogenesis of PTC.

## Figures and Tables

**Table 1:** Primer sequences of miRNA machinery components used in quantitative PCR.

Components	Position	Sequence
Dicer	Forward	5′-TTAACCTTTTGGTGTTTGATGAGTGT-3′
	Reverse	5′-AGGACATGATGGACAATT-3′
Drosha	Forward	5′-CTGTCGATGCACCAGATT-3′
	Reverse	5′-TGCATAACTCAACTGTGCAGG-3′
AGO2	Forward	5′-TCATGGTCAAAGATGAGATGACAGA-3′
	Reverse	5′-TTTATTCCTGCCCCCGTAGA-3′
DGCR8	Forward	5′-CAAGCAGGAGACATCGGACAAG-3′
	Reverse	5′-CACAATGGACATCTTGGGCTTC-3′
*β*-Actin	Forward	5′-CAGCCATGTACGTTGCTATCCAGG-3′
	Reverse	5′-AGGTCCAGACGCAGGATGGCATG-3′

**Table 2:** Correlation of the clinicopathological parameters with Dicer, Drosha, AGO2, and DGCR8 mRNA expression levels in Asian Korean PTC specimens.

	Dicer		*P*	Drosha		*P*	DGCR8		*P*	AGO2		*P*
	Low	High		Low	High		Low	High		Low	High	
Age			0.779^a^			0.310^c^			0.297^a^			0.745^c^
<40	9	5		7	7		12	2		8	6	
41–50	10	1		8	2		7	3		7	3	
>50	9	6		9	7		11	5		11	5	
T stage			0.120^a^			0.161^a^			0.170^a^			0.455^a^
(1)	16	5		14	7		14	7		12	9	
(2)	12	5		10	7		14	3		13	4	
(3)	0	2		0	2		2	0		1	1	
N stage			0.369^b^			0.121^c^			0.855^c^			0.816^c^
(+)	12	7		9	10		14	5		12	7	
(−)	16	5		15	6		16	5		14	7	
M stage			1.000^b^			0.400^b^			1.000^b^			1.000^b^
(+)	1	0		0	1		1	0		1	0	
(−)	28	12		24	15		29	10		25	14	
Histologic stage			0.562^a^			0.215^a^			0.609^a^			
(i)	13	3		11	5		13	3		10	6	
(ii)	3	2		4	1		3	2		4	1	
(iii)	4	5		4	5		7	2		5	4	
(iv)	8	2		5	5		7	3		7	3	
Extrathyroidal extension			0.716^b^			0.503^b^			0.124^b^			0.750^c^
(+)	18	9		15	12		18	9		18	9	
(−)	10	3		9	4		12	1		8	5	
Bilateral lobe involvement			0.677^b^			0.690^b^			0.388^b^			0.416^b^
(+)	5	3		4	4		5	3		4	4	
(−)	23	9		20	12		25	7		22	10	
Multifocality			1.000^b^			0.094^b^			1.000^b^			0.608^c^
(+)	11	4		6	9		11	4		9	6	
(−)	17	8		18	7		19	6		17	8	

^a^Linear by linear association.

^b^Fisher's exact test.

^c^Pearson's Chi-squared test.

**Table 3:** Correlation of the clinicopathological parameters with Dicer, Drosha, AGO2, and DGCR8 mRNA expression levels in white American patients of the TCGA.

	Dicer		*P*	Drosha		*P*	DGCR8		*P*	AGO2		*P*
	Low	High		Low	High		Low	High		Low	High	
Age			0.921^a^			0.641^a^			0.877^a^			0.507^a^
<40	9	7		10	6		8	8		11	5	
41–50	5	3		3	5		3	5		4	4	
>50	4	3		4	3		4	3		4	3	
Histologic Stage			0.901^a^			0.743^a^			0.595^a^			0.434^a^
(i)	13	8		12	9		10	11		14	7	
(ii)	2	3		2	3		2	3		2	3	
(iii)	2	2		3	1		2	2		3	1	
(iv)	1	0		0	1		1	0		0	1	
T stage			0.689^a^			0.444^a^			0.489^a^			0.187^a^
(1)	5	2		4	3		3	4		3	4	
(2)	7	6		8	5		6	7		7	6	
(3)	5	5		5	5		5	5		9	1	
(4)	1	0		0	1		1	0		0	1	
N stage			1.000^b^			0.269^b^			0.143^b^			0.264^c^
(+)	9	6		7	8		9	6		11	4	
(−)	9	6		10	5		5	10		7	8	
M stage			1.000^c^			0.433^c^			1.000^c^			0.414^c^
(+)	1	0		0	1		1	0		0	1	
(−)	16	12		17	12		14	14		17	11	
Multifocality			0.621^c^			0.607^c^			1.000^c^			0.605^c^
(+)	3	1		3	1		2	2		3	1	
(−)	15	12		14	13		13	14		16	16	

^a^Linear by linear association.

^b^Chi-squared test.

^c^Fisher's Exact test.

## Data Access

The primary and processed data used to generate the analyses presented here can be downloaded by registered users from The Cancer Genome Atlas at https://gdc-portal.nci.nih.gov/.
